# Supplementary material for: New Role for Growth/Differentiation Factor 15 in the Survival of Transplanted Brown Adipose Tissues in Cooperation with Interleukin-6
Source: Cells. 2020 Jun 1;9(6):1365. doi: 10.3390/cells9061365 (PMC7349565; doi:10.3390/cells9061365)
Supplement: Supplementary file 1 [file cells-09-01365-s001.pdf]

Supplementary information

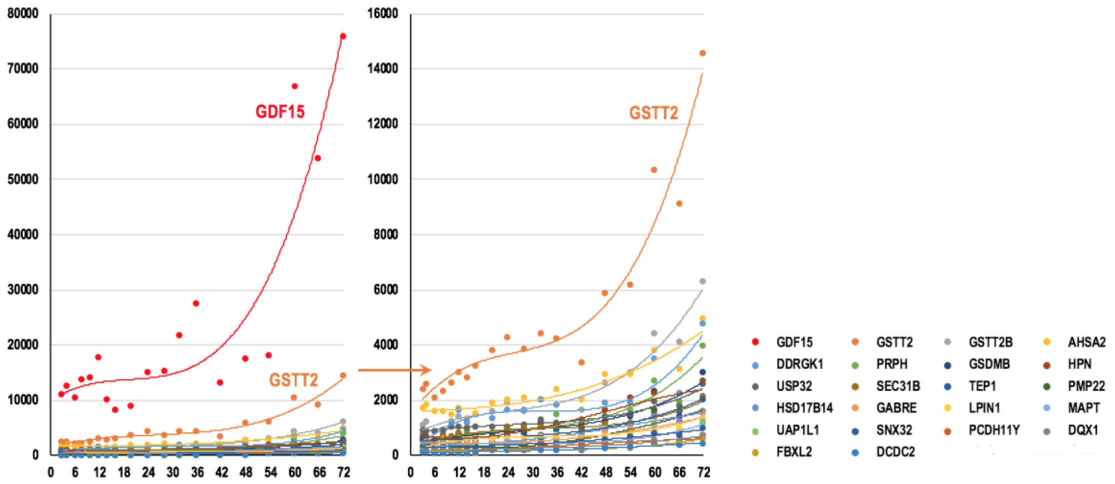

Supplementary Figure S1. Genes with monotonically increasing expressions in the earliest phase (< 72 h) of the differentiation of hESCs into BA.

Multi-time point microarray analyses were performed and genes that showed simply increasing expressions were selected. Twenty-two genes including *GDF15* showed monotonically increasing expressions. The cubic polynomial approximation curve was shown on the graph.

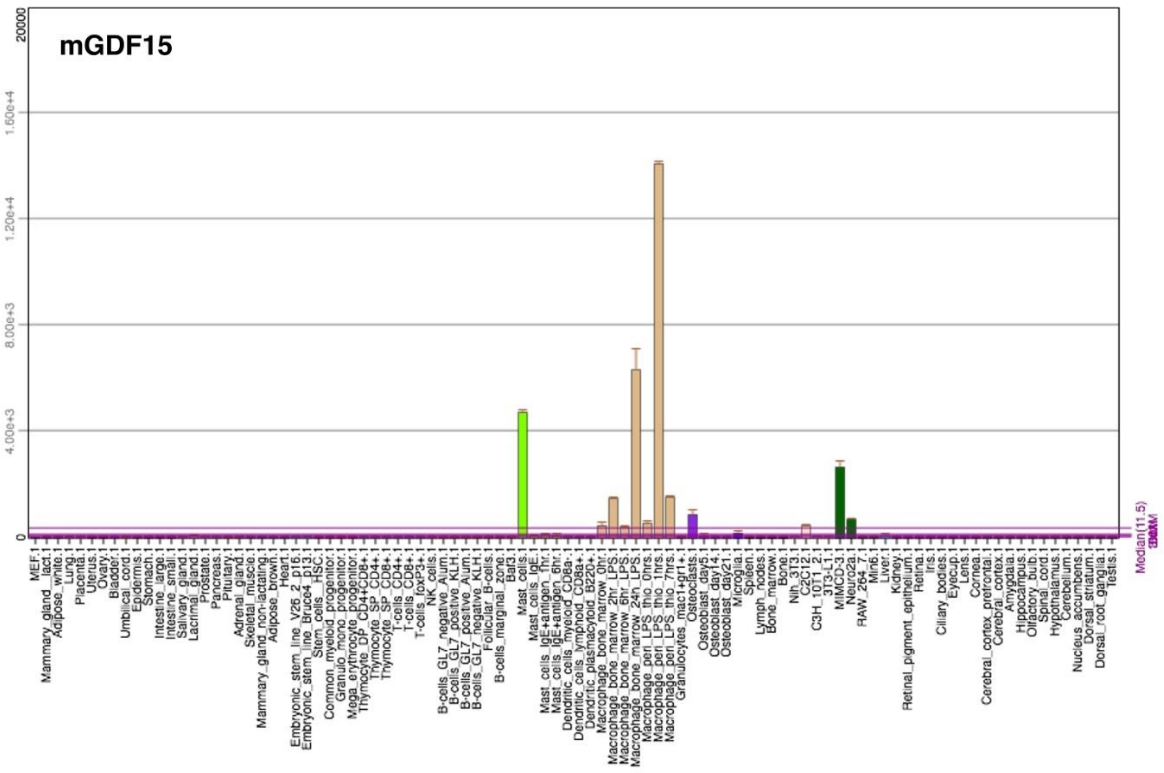

(a)

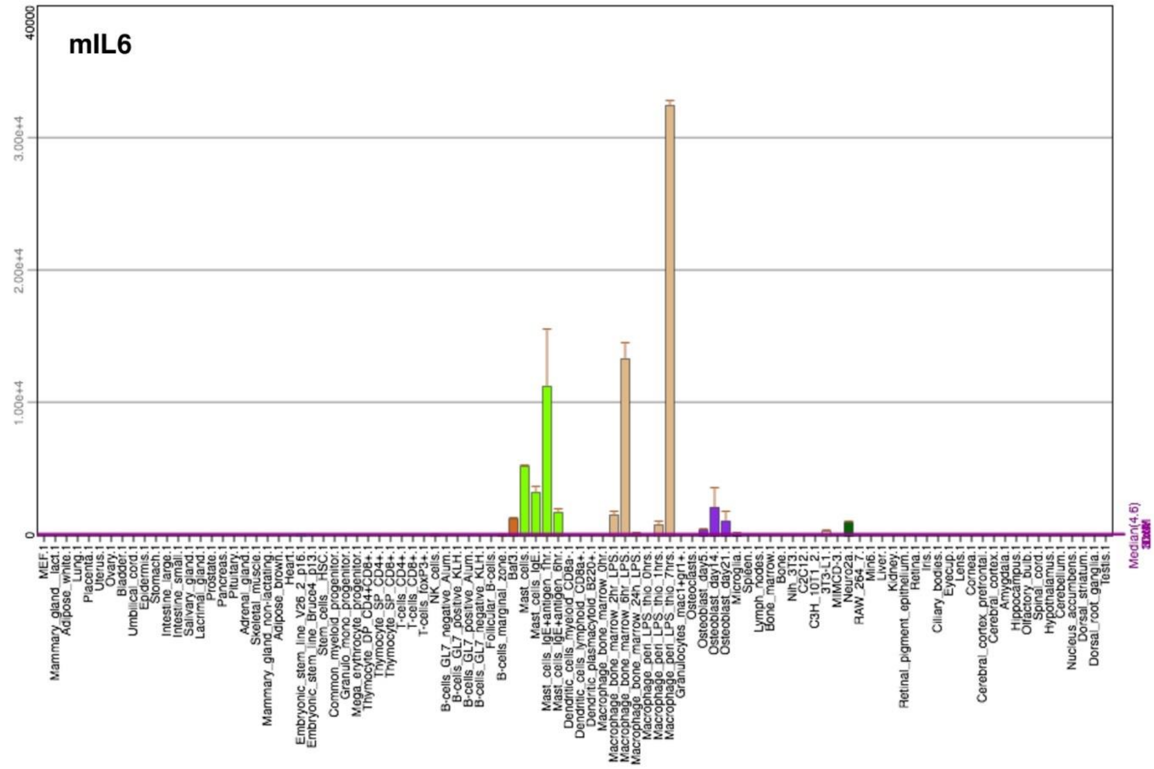

(b)

Supplementary Figure S2. High Similarity in the gene expression profile between *GDF15* and *IL6*. Tissue distribution patterns were checked by using an open database BioGPS (http://biogps.org).
URL for the data for murine *GDF15* is http://biogps.org/#goto=genereport&id=23886
URL for the data for murine *IL6* is http://biogps.org/#goto=genereport&id=16193

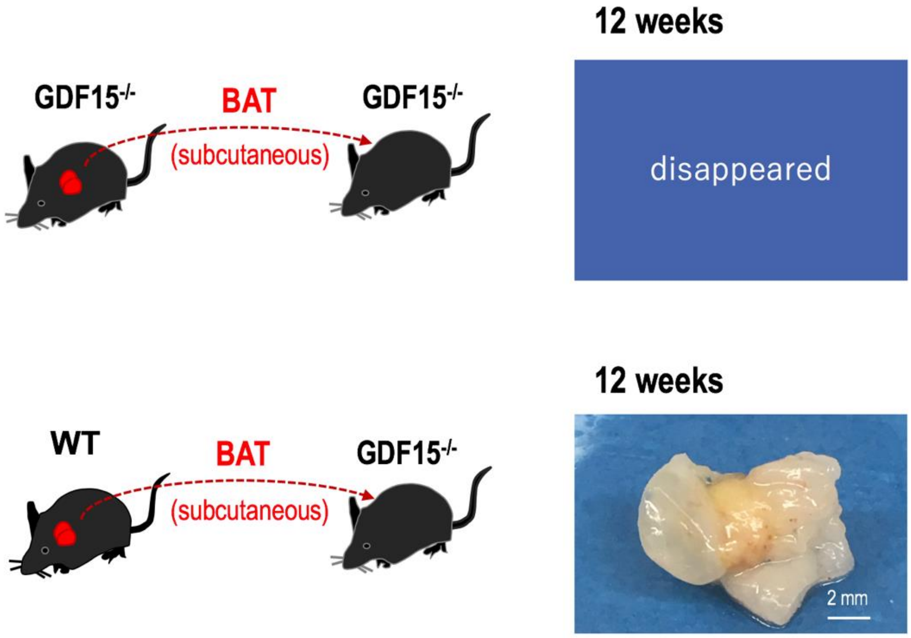

Supplementary Figure S3. GDF15 is required for long-term survival of BAT grafts.

The iBATs of either *GDF15*$^{-/-}$ or WT mice were subcutaneously transplanted into *GDF15*$^{-/-}$ mice. After 12 weeks, the grafts were removed and macroscopically observed. We could not find he BAT graft of *GDF15*$^{-/-}$ mice (upper). The BAT graft of WT mice was found with a shrunken appearance with limited neovascularization (lower).

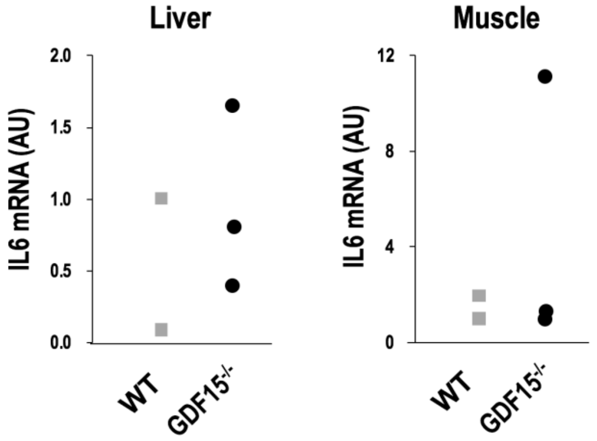

Supplementary Figure S4. IL6 expression in other tissues than BAT.
IL6 mRNA expression was measured by qRT-PCR in the liver and skeletal muscles of WT (n=2) and *GDF15*$^{-/-}$ mice (n=3). In contrast to BAT, where *IL6* expression was significantly reduced in *GDF15*$^{-/-}$ mice, the tendency *of IL6* reduction by the absence of *GDF15* was not observed in these tissues.

gtgagtagcaaccagggacccacttcaggaacaccctctccaagctccaaccaacacactcaagtccacgagcccagcccagcttccgagactcagtttcccctgctgtgacctc

tggagctgccagcagcgggctcagccagagtaggacggatgaccgtccctagaccggagctggacaaatgatgtccagagaggtcaaggtcgctcctggagatgcacagc

actcgagttggggaggacatgggggaaggttagagggtcctggagggaagagggagggacaggagccaggttgggagccaaacggaaagaacctgcgggaactgga

tcggaaatctgacgcaatcgcttgtaaagagactgctacagtaacaagtgacagtcgcttggatgggagggaggaggatccaggggactggactcagctctgggacccagc

agggctgagggcgggcccaggctgatttgggtcacgccccttacaaatccgctagggttgtgtgaatccaagcgaactgtagctctccctaaagatctgcaaagctacacttg

gatgtggtggcgcatgtctgccatcctaactactccgggggttgaagcagaattgccttgagttcaaggccagctgggactacagagtgagaacctccccaaattttatttgtag

cccaggtcgatttcaaactcagggcaatccttctgcctccatttcccaagttctgggaagacaggtgtaggcaaccaaaccctgctttaaagaaaaccaacttctgtttgttaaaca

ctttattcagaggcttgggaaaaggttggagagatccatgctgtcctgcgcagggcagaccttagggatctctgtcgctaagagcaaaatcaactttaagccagaaggtggcg

gtgtacgtctttaatcccagcacgagggtttggggtgggggtgaggggtggcacagaacaggcaaatatctgtgagttcaaggccagcctggcctacatggagagttaccag

gacagccagggctatactgagagacctatgtttgacatctgtgcagaccctgtggtcggcgggagtggcacatttgcacaggggcacatctgtgcctggcacaccctaaggac

atgagtaaccggtgttctggtttcttctgcagTGAGATTGGGGTCCCACGGCCAGCTGCTACTCCGCGTCAACCGGGCGTCGCTGA

GTCAGGGTCTCCCCGAAGCCTACCGCGTGCACCGAGCGCTGCTCCTGCTGACGCCGACGGCCCGCCCCTGGGA

CATCACTAGGCCCCTGAAGCGTGCGCTCAGCCTCCGGGGACCCCGTGCTCCCGCATTACGCCTGCGCCTGACG

CCGCCTCCGGACCTGGCTATGCTGCCCTCTGGCGGCACGCAGCTGGAACTGCGCTTACGGGTAGCCGCCGGCA

GGGGGCGCCGAAGCGCGCATGCGCACCCAAGAGACTCGTGCCCACTGGGTCCGGGGCGCTGCTGTCACTTGG

AGACTGTGCAGGCAACTCTTGAAGACTTGGGCTGGAGCGACTGGGTGCTGTCCCCGCGCCAGCTGCAGCTGAG

CATGTGCGTGGGCGAGTGTCCCCACCTGTATCGCTCCGCGAACACGCATGCGCAGATCAAAGCACGCCTGCAT

GGCCTGCAGCCTGACAAGGTGCCTGCCCCGTGCTGTGTCCCCTCCAGCTACACCCCGGTGGTTCTTATGCACAG

GACAGACAGTGGTGTGTCACTGCAGACTTATGATGACCTGGTGGCCCGGGGCTGCCACTGCGCTTGAGCACCG

GGCCCTGCTCCTCACCTACACTCCCCTTCAAGGATGCTATTTATATTTGTATTTATTAATATTATTAATTTATTGG

GGTCGGGCTGGGTGGATGGATTGTGTATTTATTTAAAACTCTGCTAATAAAGGTGAGCTTGGTTTCTataggcgtctca
gtgaccttggcatgatacccacctccctagcctcaggttccctagtgacctgagaagaggaacagaagacaggcttccattagtccggcctcaaggagattttattgactgacag
ctgatgtgggagggaccagccagtacccggtggtcctgggtgtttgagcagcaaggaggagggaggcagtttgcagctcatctctacagcctctgcttcagctcccgcctagg
tttccccagaaagtcgccttcctcccatcacgccttcaaaccaggcctaacatcccagaattagggggcctctgactaggttggggtttaagggaacctggaacagcttgaggt
gtcataacttcttgaaacacacaaaactctggctctgggtcttaatattattagtcagggttctctagattcgcagtactcatagaatgaatctatatacatatatacacacacgatat
atatatcctctagagatctagagagggagtgttattggaataaattgtaggctgtagtcgagattattcaacaatagttagctgtgagtgaaaagtgtaagaatccagtagttgct
ctgtctacaagactgggtatctcagctagtcttcagtatgtgctggagtcttgaagtaggctccagtgccagtgaaggaatgg

(a)

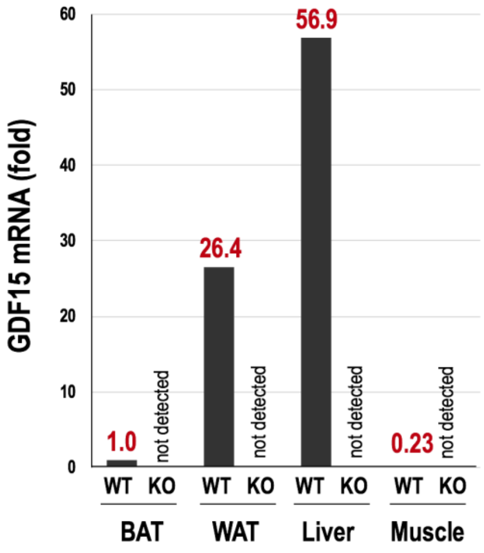

(b)

Supplementary Figure S5. Genomic DNA sequence of *GDF15$^{-/-}$* mice.

(a) A part of Intron 1 (small characters), whole sequence of Exon2 (large characters highlighted in gray) and a part of Intron 2 (small characters). DNA sequences used for constructing an expression vector for single-guide RNAs were highlighted in light blue (5′-GCTGCTACTCCGCGTCAACCGGG-3 and 5′-TATGATGACCTGGTGGCCCGGGG-3. Genomic PCR primers were shown in green characters (forward primer: 5′-caaatccgctagggttgtgt-3′; reverse primer: 5′-aacatcccagaattaggggg-3′). The deleted DNA sequences in *GDF15* knockout mice are written in blue characters (totally 874bp). The underlined sequence within Exon 2 indicates the mature peptide of *GDF15*. **(b)** Expression of *GDF15* mRNA in various insulin target tissues in *GDF15$^{-/-}$* mice. Expression levels of *GDF15* in those tissues were rather low even in WT mice, *GDF15$^{-/-}$* mice completely undetectable *GDF15* expression.

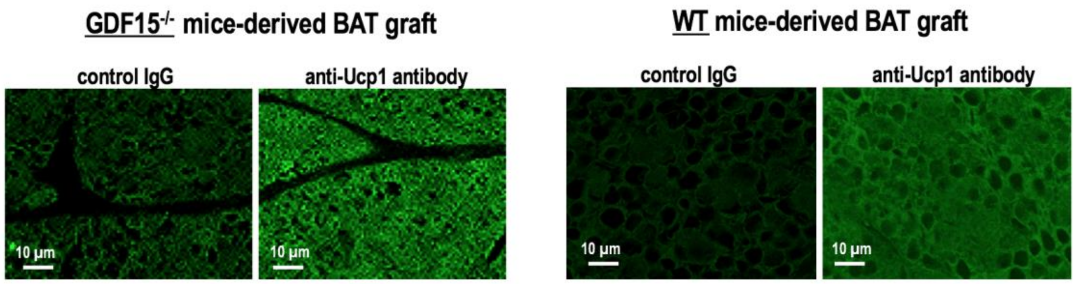

Supplementary Figure S6. Ucp1 protein expression in BAT grafts.

The intraperitoneally transplanted BAT grafts were removed after 12 weeks (Figure 8) and subjected to immunostaining using control IgG or anti-Ucp1 antibody as indicated. Reflecting the death of parenchymal cells, *GDF15*$^{-/-}$ mice-derived BAT grafts showed autofluorescence. Nevertheless, anti-Ucp1 antibody staining showed higher fluorescence activity, indicating that Ucp1 protein immunoreactivity exists in *GDF15*$^{-/-}$ mice-derived BAT grafts.
